# Intraoperative Fluorescent Imaging with Indocyanine Green during Thoracoscopic Esophagectomy with Subcarinal Lymph Node Dissection for Esophageal Cancer with a Right Superior Pulmonary Vein Anomaly: A Case Report and Literature Review

**DOI:** 10.5761/atcs.cr.25-00015

**Published:** 2025-02-27

**Authors:** Naoto Ujiie, Takanobu Nakamura, Takahiro Heishi, Yusuke Taniyama, Takashi Kamei

**Affiliations:** 1 Department of Surgery, Sendai City Hospital, Sendai, Miyagi, Japan; 2 Division of Surgery, Tohoku University Hospital, Sendai, Miyagi, Japan

**Keywords:** esophageal cancer, fluorescence imaging, indocyanine green, pulmonary vein anomaly, thoracoscopic esophagectomy

## Abstract

A 68-year-old woman was diagnosed with clinical T3N1M0 middle thoracic esophageal cancer. Preoperative three-dimensional computed tomography indicated a right superior posterior pulmonary vein (RSPPV) anomaly, which ran behind the right intermediate bronchus. The patient underwent thoracoscopic esophagectomy with mediastinal lymph node (LN) dissection. Before we began the dissection of the right subcarinal LN, we administered indocyanine green intravenously to confirm the running position of the anomalous RSPPV, and we were able to ascertain its placement accurately with correct recognition of the difference between the blood vessels and surrounding tissue. Although the patient had LN metastasis adjacent to this anomalous vessel and the dissection procedure was tough due to tightly adhesion, intraoperative fluorescent imaging enabled us to perform the dissection without any superfluous vascular injury. Intraoperative fluorescent imaging is very useful in such cases, providing accurate intraoperative information on the location of the anomaly and facilitating safer surgery.

## Introduction

The right superior posterior pulmonary vein (RSPPV), also known as the V2, usually running along the inside of the upper right lobe and merges with the right superior pulmonary vein (RSPV).^1)^ V2 anomalies are rare and one of the anomalies of the V2 runs behind the right main or intermediate bronchus and flows into the RSPV or left atrium, and its incidence is between 0.3% and 9.3%.^2)^ Preoperative identification of this anomaly is important in esophagectomy with subcarinal lymph node (LN) dissection.^3)^ If injury to an anomalous V2 occurs, it can cause major bleeding and can be life-threatening.

Previous reports of esophagectomy for esophageal cancer in patients with RSPV anomalies have focused on the importance of preoperative diagnosis of the anomaly using high-resolution imaging such as three-dimensional computed tomography (3D CT).^3–7)^ However, none of these studies have discussed the necessary surgical procedures to ensure safe esophagectomy in patients with RSPV anomalies, especially with subcarinal LN dissection. To address this gap in the literature, we describe a case in which fluorescence imaging (FI) techniques were used to verify the location of the anomalous vessel intraoperatively. This is the first report to describe a specific strategy for esophagectomy with subcarinal LN dissection in patients with RSPPV anomalies.

## Case Report

**Fig. 1:**
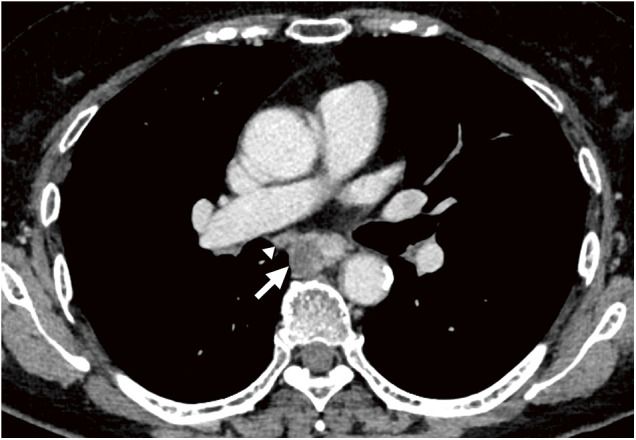
CT findings of primary lesion before neoadjuvant chemotherapy treatment. CT shows a thick wall around the middle thoracic esophagus (arrow) and enlargement of subcarinal LNs suspected of metastasis (arrowhead). CT: computed tomography; LN: lymph node

**Fig. 2:**
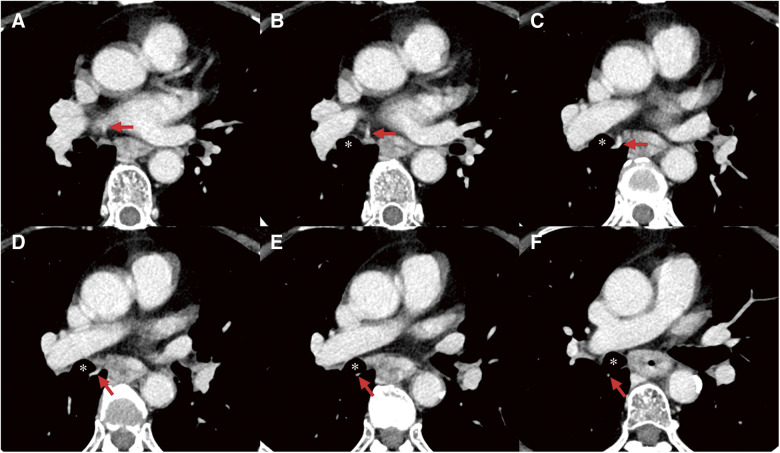
CT imaging of the course of the anomalous RSPPV, V2. (**A, B**) Anomalous RSPPV draining into RSPV. (**C–F**) Anomalous RSPPV (arrows) running dorsal side of the right intermediate bronchus. CT: computed tomography; RSPPV: right superior posterior pulmonary vein; RSPV: right superior pulmonary vein. *Right main bronchus

**Fig. 3:**
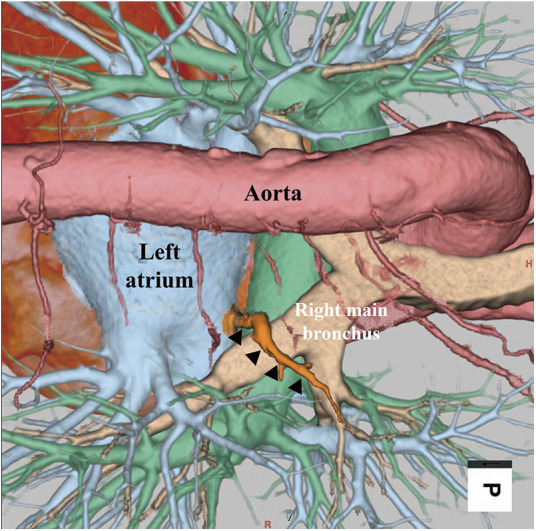
3D-CT imaging. The anomalous RSPPV (arrowheads) runs behind the right intermediate bronchus and flows into RSPV. RSPPV: right superior posterior pulmonary vein

A 68-year-old woman was treated at our hospital for Hashimoto’s disease. During a regular checkup, she complained of dysphagia. Upper gastrointestinal endoscopy revealed a type II tumor, and an esophagogram showed an irregularly shaped tumor with a 3-cm longitudinal diameter in the middle thoracic esophagus. An endoscopic biopsy indicated squamous cell carcinoma of the esophagus. CT revealed a thick wall around the middle thoracic esophagus and enlargement of the subcarinal LNs, which was suspected to be metastasis (**Fig. 1**). An RSPPV anomaly was also identified on the 3D CT. This ran behind the right intermediate bronchus from the right upper lobe into the RSPV (**Figs. 2** and **3**), and the metastatic LN appeared to be involving this vein. The patient’s blood test findings were normal including testing for squamous cell carcinoma antigens. Using the TNM classification system,^8)^ the patient was clinically diagnosed with T3N1M0, stage III squamous cell carcinoma of the esophagus.

The patient opted for surgical treatment with neoadjuvant chemotherapy for the lesion. Two cycles of cisplatin (80 mg/m^2^, day 1 and 29) and 5-fluorouracil (800 mg/m^2^, day 1–5 and 29–33) were administrated intravenously. Surgery was performed 5 weeks after completion of the chemotherapy.

**Fig. 4:**
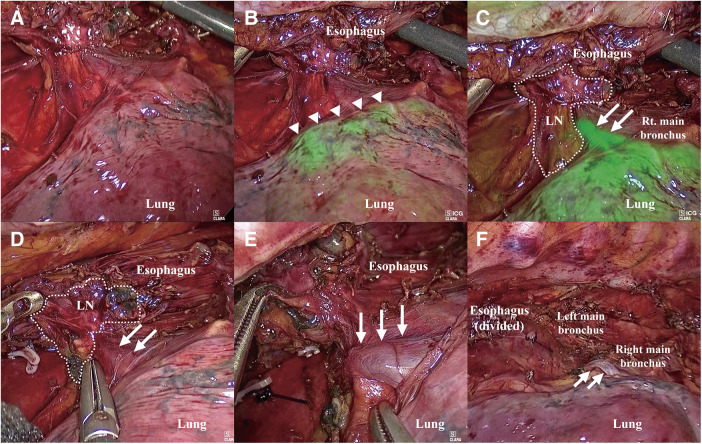
Intraoperative findings. (**A**) Uncertain boundary between the RSPPV and surrounding tissue. (**B**) The lung parenchyma was stained by green color (arrowheads) after administration of ICG. (**C**) Colorized RSPPV (arrows). (**D**) Dissection between the RSPPV (arrows) and surrounding LNs (dotted circle). (**E**) Preserved RSPPV (arrows). (**F**) Situation at the end of subcarinal LN dissection. RSPPV (arrows) was perfectly preserved and subcarinal LNs were totally dissected. LN: lymph nodes; RSPPV: right superior posterior pulmonary vein

With the patient generally anesthetized and placed in the prone position, we performed thoracoscopic esophagectomy with mediastinal LN dissection. Artificial pneumothorax was induced for the procedure using 8–10 mmHg of carbon dioxide. Prior to subcarinal LN dissection, we found that the boundary between the RSPPV and surrounding tissue was unclear (**Fig. 4A**). Therefore, we administered 10 mg of indocyanine green (ICG) intravenously to confirm the course of the RSPPV using FI. The lung parenchyma and the RSPPV were stained green approximately 30 seconds after ICG administration (**Figs. 4B** and **4C**). Henceforth, it was easy to distinguish the blood vessel from the surrounding tissue, including that of the bronchial membrane and LNs (**Fig. 4D**). Although the metastatic LNs were tightly adherent to the RSPPV and subcarinal LN dissection took around 45 min, we could then safely perform this dissection with no unnecessary injury and preservation of the RSPPV (**Figs. 4E** and **4F**). After the thoracic procedure, the patient was moved into the supine position. We performed digestive reconstruction using a gastric tube via the retrosternal route, and hand-sewn anastomosis was performed through the neck incision site.

The operative time was 586 min, and the estimated blood loss was 75 mL. Oral intake was reinitiated on postoperative day 7. Although the neck surgical wound was found to be infected, no anastomotic leakage was found, and she was discharged on postoperative day 23. A histological examination of a resected specimen revealed squamous cell carcinoma of the esophagus, with a definitive diagnosis of pT3N1M0, pStage III. Pretracheal LN metastasis was identified after 3 years of surgery, and the patient is currently undergoing treatment with the immune checkpoint inhibitor nivolumab.

## Discussion

The RSPV drains the right upper and middle lobes of the lung, with a branch usually running along the inside of the upper right lobe.^1)^ However, variations can occur in the anatomy of the pulmonary vein. An RSPV anomaly first reported in 1984 that runs along the dorsal side of the right main or intermediate bronchus.^9)^ This abnormality is an accessory vein that drains into the left atrium or RSPV separately from the ipsilateral superior pulmonary vein. Surgical research into this RSPV anomaly has primarily been in the respiratory surgery field.^2,10)^ However, there have recently been several studies of this abnormality in the field of esophageal surgery.^3–7,11,12)^ In our patient, the V2 branch of the RSPV ran behind the right intermediate bronchus before flowing into the RSPV. Preoperative identification of RSPV anomalies is critical before esophagectomy to prevent unanticipated vascular injury during surgery.

Injury of the pulmonary vein causes massive hemorrhaging. It is well-known that extensive intraoperative bleeding prevents the maintenance of an adequate operative field of view, which interferes with the continuation of the procedure, particularly during endoscopic surgery.^13)^ Furthermore, the reconstruction of an injured pulmonary vein can result in insufficient drainage of venous blood due to stenosis. This may induce partial pulmonary congestion and hemoptysis.^6)^ In some instances, injury to the pulmonary vein can be fatal. Therefore, any RSPV abnormalities must be identified preoperatively. This facilitates the avoidance of injury to the vein by allowing accurate planning of a surgical procedure that optimizes safety and efficacy.

There have been seven previous reports of esophagectomy for esophageal cancer with an RSPV anomaly in English academic literature (**Table 1**).^3–7,11,12)^ Most of these emphasize the importance of preoperative identification of the anomalous vein using high-resolution CT to prevent vascular injury. In two of the reports, the anomalous RSPV was not identified preoperatively but was noticed intraoperatively.^11,12)^ In the other four cases, the RSPV anomaly was diagnosed preoperatively by 3D CT.^3–7)^ However, none of these studies indicate how such anomalies should be managed during esophagectomy with subcarinal LN dissection. Although technological advances in diagnostic imaging such as 3D CT allow detailed preoperative visualization of regional anatomy, it can sometimes be difficult to correctly identify the parts of the image within the patient’s anatomy during an operation because of abundant adipose tissue or thickened membranes. This can result in misidentification of tissue and unnecessary damage, such as vascular injury. In the present case, we identified the anomalous V2 preoperatively and decided in advance on the measures needed to avoid vascular injury.

**Table 1:** Review of previous English literature about esophagectomy with an RSPV anomaly

Case	Author	Year	Age	Sex	Running location of anomalous RSPV	Origin of anomalous RSPV	Inflow	Preoperative recognition	Operative procedure	Reconstruction route	Blood loss (mm)	Preservation (Yes/No)
1	Matsubara^11)^	2003	57	Male	Intermediate bronchus	Right upper lobe	LA	No	Thoracotomy	–	–	Yes
2	Shiozaki^12)^	2016	74	Male	Main bronchus	Right upper lobe	–	No	Transhiatal approach	RR	169	Yes
3	Onodera^3)^	2019	61	Male	Intermediate bronchus	S2	LA	Yes	Thoracoscopy	PMR	52	Yes
4	Matsubara^4)^	2020	77	Male	Intermediate bronchus	S2	RSPV	Yes	Thoracoscopy	PMR	150	Yes
5	Sato^5)^	2022	70	Male	Main bronchus	S2	RSPV	Yes	Thoracoscopy	RR	30	Yes
6	Kuwayama^6)^	2022	81	Male	Main bronchus	Right upper lobe	LA	Yes	Thoracoscopy	–	–	Yes
7	Mikami^7)^	2023	60	Male	Main bronchus	Right upper lobe	LA	Yes	Thoracoscopy	RR	45	Yes
8	Our case		68	Female	Intermediate bronchus	S2	RSPV	Yes	Thoracoscopy	RR	75	Yes

RSPV: right superior pulmonary vein; S2: posterior segment of right upper lobe; LA: left atrium; RR: retrosternal route; PMR: posterior mediastinal route

Intraoperative FI with ICG is established as a convenient and safe surgical tool, having previously been used in the surgical treatment of gastrointestinal cancer, breast cancer, gynecological cancer, and transplantation surgery.^14–18)^ Intraoperative FI with ICG allows real-time visualization of the anatomy of the blood supply and lymphatic drainage system in the operative area. In esophagectomy, intraoperative FI with ICG is commonly used to assess gastric conduit perfusion and for the prevention of thoracic duct injuries.^19)^ In a patient with an anomalous RSPPV, we applied this approach to verify the running position of the pulmonary vein. Based on this experience, we are confident that the dissection procedure can be performed on the correct layer with clear distinctions between the blood vessels and surrounding tissue, including the bronchus and LNs. Additionally, the ICG staining remains visible for a while, allowing for repeated confirmation of the course of the RSPPV using the ICG scope whenever necessary. This optimizes operative outcomes and prevents injury to the pulmonary vein, especially in the case which metastatic LN involving this anormal RSPPV. FI is a useful means of intraoperative confirmation of the vascular location during esophagectomy with mediastinal dissection, leading to safe surgical success in patients with RSPV anomalies.

## Conclusion

To the best of our knowledge, this is the first report to discuss the intraoperative plan for esophagectomy with subcarinal LN dissection in patients with an anomalous V2. In patients with esophageal cancer and an RSPV anomaly receiving thoracoscopic esophagectomy with mediastinal LN dissection, intraoperative FI with ICG is a useful tool. It allows the surgeon to identify the correct vascular location in real time. This results in safer surgery and the prevention of superfluous vascular injury.

## Declarations

### Ethics approval and consent to participate

This study was approved by the Sendai City Hospital (20230109), and written informed consent to participate was obtained from the patient.

### Consent for publication

Written informed consent was obtained from the patient for publication of this case report.

### Funding

None of authors received any funding.

### Disclosure statement

The authors declare that they have no competing interests.

### Data availability

The data that support the findings of this study are available from the corresponding author upon reasonable request.

### Author contributions

NU made substantial contributions to the concept and design of the case report.

TN, TH, and YT participated in the operation of this case.

TN and TH treated the patient after the operation.

YT and TK supervised the operation. NU drafted the manuscript.

TN, TH, YT, and TK edited the manuscript.

All authors read and approved the final manuscript.
